# Supplementary material for: Anti-Inflammatory Drug Therapy in Chronic Subdural Hematoma: A Systematic Review and Meta-Analysis of Prospective Randomized, Double-Blind and Placebo-Controlled Trials
Source: Int J Mol Sci. 2022 Dec 19;23(24):16198. doi: 10.3390/ijms232416198 (PMC9784956; doi:10.3390/ijms232416198)
Supplement: Supplementary file 1 [file ijms-23-16198-s001.zip › ijms-2053943-supplementary.pdf]

Supplementary Material

Supplementary Table S1. Characteristics of excluded prospective trials on anti-inflammatory studies in patients with chronic subdural hematoma.

Name	Year	Blinding	Treatment	No. patients total	No. patients in treatment	No. patients in control	Cause of exclusion
Tariq et al. [33]	2021	Single-blinded	Dexamethasone	92	46	46	Single-blinded, no placebo control
Fujisawa et al. [34]	2021	Not blinded	Goreisan	208	104	104	No placebo control
Katayama et al. [35]	2018	Not blinded	Goreisan	180	88	92	No placebo control
Workewych et al. [36]	2018	Single-blinded	Tranexamic acid	24	NA	NA	No anti-inflammatory drug
Yamada and Natori [37]	2020	Not blinded	Tranexamic acid and Goreisan	193	150	43	No anti-inflammatory drug, no placebo control
Chan et al. [38]	2015	Not blinded	Dexamethasone	248	122	126	No placebo control
Sun et al. [39]	2005	Not blinded	Dexamethasone	112	95	17	No placebo control
Hirashima et al. [40]	2002	Not blinded	Etizolam	53	24	29	No placebo control
Hirashima et al. [41]	2002	Not blinded	Etizolam	39	15	24	No placebo control
Schaumann et al. [42]	2016	Not blinded	Celecoxib	23	10	13	No placebo control

SUPPLEMENTARY TABLE S2: Quality assessment of prospective randomized double-blind, and placebo-controlled trials		
1. Prud´homme 2016 [32]	Risk of bias	Author judgment

Random sequence generation (selection bias)	Low-risk	The method of randomization is reported. Web-based service by pharmacist who was involved in the study.
Allocation concealment (selection bias)	Low-risk	Allocation to each group in a 1:1 ratio with block sizes ranging from 4 to 6.
Blinding of participants and personnel (performance bias)	Low-risk	Double blinded
Blinding of outcome assessment (detection bias)	Low-risk	Double blinded
Incomplete outcome data (attrition bias)	Low risk	Data is recorded for all patients
Selective reporting (reporting bias)	Low risk	All prespecified outcomes were reported
Other bias	Unclear	
2. Jiang 2018 [31]	Risk of bias	Author judgment
Random sequence generation (selection bias)	Low risk	The method of randomization is reported. Patients were centrally randomized using the Data Acquisition System for Electronic Data Capture (Version 5.0).
Allocation concealment (selection bias)	Low risk	Allocation to each group in a 1:1 ratio.
Blinding of participants and personnel (performance bias)	Low risk	Double blinded
Blinding of outcome assessment (detection bias)	Low risk	Double blinded
Incomplete outcome data (attrition bias)	Unclear risk	Exclusion of several patients in both arms because of refusal of CT scans or medication, lost to follow-up, withdrawal of the decision, and death. Modified intention-treat analysis was performed.
Selective reporting (reporting bias)	Low risk	All prespecified outcomes were reported.
Other bias	Unclear	
3. Mebberson 2019 [30]	Risk of bias	Author judgment
Random sequence generation (selection bias)	Low risk	The method of randomization is reported. Computer-derived permuted block with varying block size sequence was used for randomization.
Allocation concealment (selection bias)	Low risk	Allocation to each group in a 1:1 ratio.
Blinding of participants and personnel (performance bias)	Low risk	Double blinded

Blinding of outcome assessment (detection bias)	Low risk	Double blinded
Incomplete outcome data (attrition bias)	Unclear	One patient of the dexamethasone arm missed the evaluation of the mRS at 6-months for unknown reason.
Selective reporting (reporting bias)	Low risk	All prespecified outcomes were reported.
Other bias	Unclear	
4. Hutchinson 2020 [29]	Risk of bias	Author judgment
Random sequence generation (selection bias)	Low risk	The method of randomization is reported. Randomization was performed with the use of permute blocks (random block sizes of two or four).
Allocation concealment (selection bias)	Low risk	Allocation to each group in a 1:1 ratio.
Blinding of participants and personnel (performance bias)	Low risk	Double blinded
Blinding of outcome assessment (detection bias)	Low risk	Double blinded
Incomplete outcome data (attrition bias)	Low risk	Multicentric trial with lost to follow-up. Intention-to-treat analysis was performed to counteract the lack of data.
Selective reporting (reporting bias)	Low risk	All prespecified outcomes were reported.
Other bias	Unclear	
5. Ng 2021 [28]	Risk of bias	Author judgment
Random sequence generation (selection bias)	Low risk	The method of randomization is reported. Block sizes of four patients were used.
Allocation concealment (selection bias)	Low risk	Allocation to each group in a 1:1 ratio.
Blinding of participants and personnel (performance bias)	Low risk	Double blinded
Blinding of outcome assessment (detection bias)	Low risk	Double blinded
Incomplete outcome data (attrition bias)	Low risk	Multicentric trial with lost to follow-up. Intention-to-treat analysis was performed to counteract the lack of data.
Selective reporting (reporting bias)	Low risk	All prespecified outcomes were reported.
Other bias	Unclear	

SUPPLEMENTARY TABLE S3: Assessment of certainty – general population

Certainty assessment							№ of patients		Effect		Certainty
**№ of studies**	**Study design**	**Risk of bias**	**Inconsistency**	**Indirectness**	**Imprecision**	**Other considerations**	**Anti-inflammatory drug therapy**	**placebo**	**Relative (95% CI)**	**Absolute (95% CI)**	
**Mortality**											
5	randomised trials	not serious	not serious	not serious	not serious	none	42/542 (7.7%)	24/540 (4.4%)	**OR 1.79** (1.06 to 3.00)	**32 more per 1 000** (from 3 more to 78 more)	⊕⊕⊕⊕ High
**Neurological outcome (follow-up: range 2 months to 72 months; assessed with: modified Rankin scale)**											
4	randomised trials	not serious	not serious	not serious	not serious	none	401/524 (76.5%)	406/521 (77.9%)	**OR 1.12** (0.63 to 2.00)	**19 more per 1 000** (from 89 fewer to 97 more)	⊕⊕⊕⊕ High
**Secondary surgery**											
5	randomised trials	not serious	not serious	not serious	not serious	none	22/558 (3.9%)	24/559 (4.3%)	**OR 0.35** (0.21 to 0.58)	**27 fewer per 1 000** (from 34 fewer to 18 fewer)	⊕⊕⊕⊕ High

CI: confidence interval; **OR:** odds ratio

SUPPLEMENTARY TABLE S4: Assessment of certainty – conservatively treated population

Certainty assessment							№ of patients		Effect		Certainty
**№ of studies**	**Study design**	**Risk of bias**	**Inconsistency**	**Indirectness**	**Imprecision**	**Other considerations**	**Anti-inflammatory drug therapy**	**placebo**	**Relative (95% CI)**	**Absolute (95% CI)**	
**Mortality**											
2	randomised trials	not serious	not serious	not serious	not serious	none	3/108 (2.8%)	1/108 (0.9%)	**OR 2.21** (0.27 to 17.92)	**11 more per 1 000** (from 7 fewer to 134 more)	⊕⊕⊕⊕ High
**Secondary surgery**											
2	randomised trials	not serious	not serious	not serious	not serious	none	12/108 (11.1%)	26/108 (24.1%)	**OR 0.40** (0.19 to 0.83)	**128 fewer per 1 000** (from 184 fewer to 32 fewer)	⊕⊕⊕⊕ High

CI: confidence interval; **OR:** odds ratio

SUPPLEMENTARY TABLE S5: Assessment of certainty – surgically treated patients

Certainty assessment							№ of patients		Effect		Certainty
**№ of studies**	**Study design**	**Risk of bias**	**Inconsistency**	**Indirectness**	**Imprecision**	**Other considerations**	**Anti-inflammatory drug therapy**	**placebo**	**Relative (95% CI)**	**Absolute (95% CI)**	
**Mortality (follow-up: range 2 to 72)**											
3	randomised trials	not serious	not serious	not serious	not serious	none	39/434 (9.0%)	23/432 (5.3%)	**OR 1.76** (1.03 to 3.01)	**37 more per 1 000** (from 2 more to 92 more)	⊕⊕⊕⊕ High
**Neurological outcome (follow-up: range 2 months to 72 months; assessed with: modified Rankin scale)**											
3	randomised trials	not serious	not serious	not serious	not serious	none	306/426 (71.8%)	315/423 (74.5%)	**OR 0.86** (0.63 to 1.17)	**30 fewer per 1 000** (from 97 fewer to 29 more)	⊕⊕⊕⊕ High
**Secondary surgery**											
3	randomised trials	not serious	not serious	not serious	not serious	none	10/450 (2.2%)	35/451 (7.8%)	**OR 0.31** (0.11 to 0.89)	**52 fewer per 1 000** (from 68 fewer to 8 fewer)	⊕⊕⊕⊕ High

CI: confidence interval; **OR:** odds ratio

SUPPLEMENTARY TABLE S6: Assessment of certainty – use of corticosteroids

Certainty assessment							№ of patients		Effect		Certainty
№ of studies	Study design	Risk of bias	Inconsistency	Indirectness	Imprecision	Other considerations	Corticosterids	placebo	Relative (95% CI)	Absolute (95% CI)	
**Mortality**											
4	randomised trials	not serious	not serious	not serious	not serious	none	41/444 (9.2%)	23/442 (5.2%)	**OR 1.83** (1.08 to 3.09)	**39 more per 1 000** (from 4 more to 93 more)	⨁⨁⨁⨁ High
**Secondary surgery**											
4	randomised trials	not serious	not serious	not serious	not serious	none	11/460 (2.4%)	38/461 (8.2%)	**OR 0.30** (0.14 to 0.63)	**56 fewer per 1 000** (from 70 fewer to 29 fewer)	⨁⨁⨁⨁ High

CI: confidence interval; **OR:** odds ratio

PRISMA 2020 Checklist

Section and Topic	Item #	Checklist item	Location where item is reported
**TITLE**			
Title	1	Identify the report as a systematic review.<br><br>**Anti-inflammatory drug therapy in chronic subdural hematoma: A systematic review and meta-analysis of prospective randomized, double-blind, and placebo-controlled trials**	Title
**ABSTRACT**			
Abstract	2	**Although the anti-inflammatory drug therapy has been identified as potentially beneficial for patients suffering from chronic subdural hematoma (cSDH), contemporary literature presents contradictory results. In this meta-analysis, we aim to investigate the impact of anti-inflammatory drug therapy on mortality and outcome. We searched for eligible randomised, placebo-controlled prospective trials (RTC) on PubMed, Embase and Medline in July 2022. From 97 articles identified initially, 5 RTCs meet the criteria and were included in our meta-analysis.**	Abstract
**INTRODUCTION**			
Rationale	3	Describe the rationale for the review in the context of existing knowledge.<br><br>**According to contemporary knowledge, inflammation is proposed as a leading factor for fluid collection and further expansion of pre-existing hematoma. Vascular endothelial growth factor (VEGF) may be secreted by inflammatory cellular components (such as neutrophils or macrophages) infiltrating the cSDH and supporting the growth. Therefore, suppression of this processes might be beneficial for patients suffering on cSDH. Up to date, many authors tried to evaluate the effect of anti-inflammatory drugs in patients with cSDH with mixed results.**	Introduction
Objectives	4	**To assess the anti-inflammatory drug therapy effect for patients with cSDH, the authors will conduct a meta-analysis of all eligible results.**	Introduction
**METHODS**			
Eligibility criteria	5	Specify the inclusion and exclusion criteria for the review and how studies were grouped for the syntheses.<br><br>**The authors conducted a systematic search of the Pubmed, Embase and Medline databases in July 2022 for the terms "chronic subdural hematoma". The search was limited to "randomized controlled trials", "human studies", "clinical trials" and "English". The literature search included all results until the 30th of June 2022. The inclusion criteria were formulated according to the PICOS (population, intervention, comparator, outcomes and study design) framework [20].These criteria were as follow: patients had undergone treatment for chronic subdural hematoma; relevant anti-inflammatory drug therapies were performed; results were compared to a placebo control and all results of the prespecified endpoints are reported; and the trials were defined as prospective randomized, placebo-controlled, and double-blinded studies. The following types of records were excluded: reviews, study protocols, letters, conference abstracts, unpublished papers, animal experiments, and trials with insufficient data (e.g., no placebo control or not double-blinded). Furthermore, previous meta-analyses and reviews were searched for studies matching our inclusion and exclusion criteria.**	Methods

PRISMA 2020 Checklist

Section and Topic	Item #	Checklist item	Location where item is reported
Information sources	6	Specify all databases, registers, websites, organisations, reference lists and other sources searched or consulted to identify studies. Specify the date when each source was last searched or consulted.<br><br>**We searched the Pubmed, Embase and Medline databases in July 2022 for the terms "chronic subdural hematoma".**	Methods
Search strategy	7	Present the full search strategies for all databases, registers and websites, including any filters and limits used.<br><br>**The search was limited to "randomized controlled trials", "human studies", "clinical trials" and "English". The literature search included all results until the 30th of June 2022.**	Methods
Selection process	8	Specify the methods used to decide whether a study met the inclusion criteria of the review, including how many reviewers screened each record and each report retrieved, whether they worked independently, and if applicable, details of automation tools used in the process.<br><br>**We performed title screening, abstract screening and in case of uncertainty a whole text screening to search for the eligible studies.**	Methods
Data collection process	9	Specify the methods used to collect data from reports, including how many reviewers collected data from each report, whether they worked independently, any processes for obtaining or confirming data from study investigators, and if applicable, details of automation tools used in the process.<br><br>**The data collection was performed by two authors independently (MV, JW). The disagreement between both reviewers was settled by third author (EG).**	Methods
Data items	10a	List and define all outcomes for which data were sought. Specify whether all results that were compatible with each outcome domain in each study were sought (e.g. for all measures, time points, analyses), and if not, the methods used to decide which results to collect.<br><br>**We analyzed the studies to conduct a meta-analysis according to following outcomes:**<br>**1) in case of postoperative administration of anti-inflammatory drugs, following outcomes were analyzed:**<br>• **mortality**<br>• **general outcome (dichotomized as good and poor)**<br>• **recurrence/revision rates**<br>**2) in case of conservative therapy with anti-inflammatory drugs, following outcomes were assessed:**<br>• **switch to surgery rates**<br>• **mortality**	Methods

PRISMA 2020 Checklist

Section and Topic	Item #	Checklist item	Location where item is reported
		• **general outcome (dichotomized as good and poor)**<br>• **recurrence/revision rates**	
	10b	List and define all other variables for which data were sought (e.g. participant and intervention characteristics, funding sources). Describe any assumptions made about any missing or unclear information.<br><br>• **Mortality was assessed according to provided data as patients, who deceased in the course of the therapy.**<br>• **We defined general outcome according to modified Ranking scale (mRS) and dichotomized into good (mRS 0-3) and poor (mRS 4-5)**<br>• **Switch to surgery was defined operative evacuation of the hematoma by patient, who was initially treated conservatively.**<br>• **Recurrence or revision rate was defined as necessity of second operative hematoma evacuation by patient, who already underwent surgical treatment.**	Methods
Study risk of bias assessment	11	Specify the methods used to assess risk of bias in the included studies, including details of the tool(s) used, how many reviewers assessed each study and whether they worked independently, and if applicable, details of automation tools used in the process.<br><br>**We used the Cochrane Bias Risk Tool to investigate the risk of bias (ROB) in the included trials using the software Review Manager Web (RevMan Web Version 5.4.1 from The Cochrane Collaboration, available at revman.cochrane.org). The following six characteristics regarding risk of bias assessment were included in the analysis: selection bias, performance bias, detection bias, attrition bias, reporting bias, and other sources of bias. Afterwards, a risk of bias summary chart and plot were created.**<br>**To explore the statistical heterogeneity and inconsistency, x^2 and I^2 statistics were used respectively; an I^2 value of 50% or more represented substantial heterogeneity. Weight to the size of each study was involved with regard to the estimation of treatment effects.**<br>**Funnel plots were used to examine the publication bias of included studies. Effect sizes were expressed as pooled OR estimates. Finally, death, recurrence and poor outcome were analysed accordingly.**	Methods
Effect measures	12	Describe the processes used to decide which studies were eligible for each synthesis (e.g. tabulating the study intervention characteristics and comparing against the planned groups for each synthesis).<br><br>**We measured and reported outcomes in Forest-plot, providing heterogeneity and inconsistency analysis, pooled odds ratio and statistical significance.**	Methods
Synthesis	13a	Describe the processes used to decide which studies were eligible for each synthesis (e.g. tabulating the study intervention	Methods

PRISMA 2020 Checklist

Section and Topic	Item #	Checklist item	Location where item is reported
methods		characteristics and comparing against the planned groups for each synthesis (item #5)).<br><br>**To see all the eligible studies and reported outcomes, see Table 1.**	
	13b	Describe any methods required to prepare the data for presentation or synthesis, such as handling of missing summary statistics, or data conversions.<br><br>**To see all the eligible studies and reported outcomes, see Table 1.**	Methods
	13c	Describe any methods used to tabulate or visually display results of individual studies and syntheses.<br><br>**For visualisation of our Meta-analysis, Forest Plot created with Review Manager Web (RevMan Web Version 5.4.1 from The Cochrane Collaboration, available at revman.cochrane.org) were presented as figures to selected outcomes.**	Methods
	13d	Describe any methods used to synthesize results and provide a rationale for the choice(s). If meta-analysis was performed, describe the model(s), method(s) to identify the presence and extent of statistical heterogeneity, and software package(s) used.<br><br>**We used the Cochrane Bias Risk Tool to investigate the risk of bias (ROB) in the included trials using the software Review Manager Web (RevMan Web Version 5.4.1 from The Cochrane Collaboration, available at revman.cochrane.org). The following six characteristics regarding risk of bias assessment were included in the analysis: selection bias, performance bias, detection bias, attrition bias, reporting bias, and other sources of bias. Afterwards, a risk of bias summary chart and plot were created.**<br>**To explore the statistical heterogeneity and inconsistency, x^2 and I^2 statistics were used respectively; an I^2 value of 50% or more represented substantial heterogeneity. Weight to the size of each study was involved with regard to the estimation of treatment effects.**<br>**Funnel plots were used to examine the publication bias of included studies. Effect sizes were expressed as pooled OR estimates. Finally, death, recurrence and poor outcome were analyzed accordingly.**<br><br>**Two methods were used to assess the publication bias. First, funnel plots were used to visually examine the publication bias of included studies. Second, Egger regression test was used to statistically investigate the funnel plot symmetry. The likelihood of publication bias was determined using the Egger regression intercept two-tailed test and a 5% significance threshold was set [23]. Third, Begg´s test was used to evaluate the asymmetry of the data [24]. Egger´s and Begg´s test were performed using MedCalc (Version 20.123 for Windows). Effect sizes were expressed as pooled OR estimates. Finally, death, recurrence and poor outcome were analyzed accordingly.**	Methods
	13e	Describe any methods used to explore possible causes of heterogeneity among study results (e.g. subgroup analysis, meta-regression).	Methods

PRISMA 2020 Checklist

Section and Topic	Item #	Checklist item	Location where item is reported
		**In case of subgroup-analysis, there might be a heterogeneity of the data caused by lack of published results.**	
	13f	Describe any sensitivity analyses conducted to assess robustness of the synthesized results.<br><br>**Not available**	Methods
Reporting bias assessment	14	Describe any methods used to assess risk of bias due to missing results in a synthesis (arising from reporting biases).<br><br>**According to our strict inclusion criteria and high quality of included studies, we do not suppose to have missing-results bias.**	Methods
Certainty assessment	15	Describe any methods used to assess certainty (or confidence) in the body of evidence for an outcome.<br><br>**For report on certainty assessment see Supplementary Tables 3-6**	Methods
**RESULTS**			
Study selection	16a	Describe the results of the search and selection process, from the number of records identified in the search to the number of studies included in the review, ideally using a flow diagram.<br>**Process of the search and selection is summarized in Figure 1.**	Figure 1
	16b	Cite studies that might appear to meet the inclusion criteria, but which were excluded, and explain why they were excluded.<br>**Excluded prospective trials are summarized in supplementary Table S1.**	Table S1
Study characteristics	17	Cite each included study and present its characteristics.<br>**Studies included are summarized in Table 1.**	Table 1
Risk of bias in studies	18	Present assessments of risk of bias for each included study.<br>**Risk of bias analysis is reported in supplementary Table S2.**	Table S2
Results of individual studies	19	For all outcomes, present, for each study: (a) summary statistics for each group (where appropriate) and (b) an effect estimate and its precision (e.g. confidence/credible interval), ideally using structured tables or plots.<br>**Analysis on every outcome is separately reported on with it´s own forest plot.**	Figure 2-15
Results of syntheses	20a	For each synthesis, briefly summarise the characteristics and risk of bias among contributing studies.<br>**Risk of bias is summarized in Funnel-Plot in each analysis separately.**	Figure 16
	20b	Present results of all statistical syntheses conducted. If meta-analysis was done, present for each the summary estimate and its precision (e.g. confidence/credible interval) and measures of statistical heterogeneity. If comparing groups, describe the direction of the effect.<br>**Presented.**	Results 3.4-3.8

PRISMA 2020 Checklist

Section and Topic	Item #	Checklist item	Location where item is reported
	20c	Present results of all investigations of possible causes of heterogeneity among study results.<br>**Presented.**	Results 3.4-3.8
	20d	Present results of all sensitivity analyses conducted to assess the robustness of the synthesized results.<br>**Presented.**	Results 3.4-3.8
Reporting biases	21	Present assessments of risk of bias due to missing results (arising from reporting biases) for each synthesis assessed.<br>**Not available.**	N/A
Certainty of evidence	22	Present assessments of certainty (or confidence) in the body of evidence for each outcome assessed.<br>**Presented.**	Supplementary Table S3
**DISCUSSION**			
Discussion	23a	Provide a general interpretation of the results in the context of other evidence.<br>**Presented.**	Discussion
	23b	Discuss any limitations of the evidence included in the review.<br>**Presented.**	Discussion, last paragraph
	23c	Discuss any limitations of the review processes used.<br>**Presented.**	N/A due to strict selection criteria
	23d	Discuss implications of the results for practice, policy, and future research.<br>**Presented.**	Discussion
**OTHER INFORMATION**			
Registration and protocol	24a	Provide registration information for the review, including register name and registration number, or state that the review was not registered.<br>**Review was not registered.**	
	24b	Indicate where the review protocol can be accessed, or state that a protocol was not prepared.<br>**Protocol was not prepared.**	
	24c	Describe and explain any amendments to information provided at registration or in the protocol.<br>**No amendments to describe.**	
Support	25	Describe sources of financial or non-financial support for the review, and the role of the funders or sponsors in the review.<br>**There is no financial support to describe.**	
Competing interests	26	Declare any competing interests of review authors.<br>**No interest to declare.**	

PRISMA 2020 Checklist

Section and Topic	Item #	Checklist item	Location where item is reported
Availability of data, code and other materials	27	Report which of the following are publicly available and where they can be found: template data collection forms; data extracted from included studies; data used for all analyses; analytic code; any other materials used in the review.<br>**Template data collection form, data extracted and used for the analysis are reported in method section. The software is used to conduct the meta-analysis is available online.**	

From: Page MJ, McKenzie JE, Bossuyt PM, Boutron I, Hoffmann TC, Mulrow CD, et al. The PRISMA 2020 statement: an updated guideline for reporting systematic reviews. BMJ 2021;372:n71. doi: 10.1136/bmj.n71

For more information, visit: http://www.prisma-statement.org/

Supplementary Figure S1. PRISMA checklist of the present meta-analysis [18].

Study or Subgroup	Experimental Events	Experimental Total	Control Events	Control Total	Weight	Odds Ratio M-H, Random, 95% CI	Year
Mebberson 2019	17	22	16	24	5.7%	1.70 [0.46, 6.30]	2019
Hutchinson 2020	226	341	240	339	93.3%	0.81 [0.59, 1.12]	2020
Ng 2021	63	63	59	60	0.9%	3.20 [0.13, 80.14]	2021
**Total (95% CI)**		**426**		**423**	**100.0%**	**0.86 [0.63, 1.17]**	
Total events	306		315				

Heterogeneity: Tau² = 0.00; Chi² = 1.81, df = 2 (P = 0.40); I² = 0%
Test for overall effect: Z = 0.97 (P = 0.33)

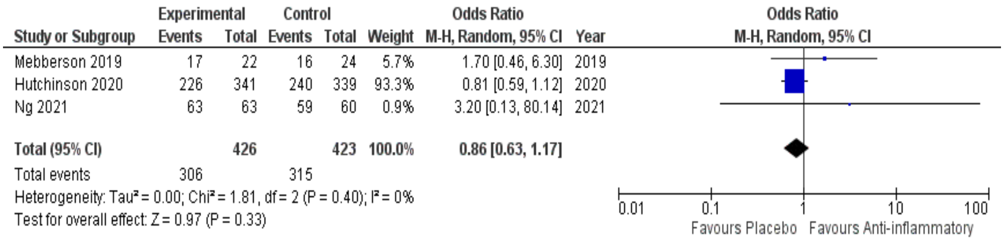

Supplementary Figure S2. Forest Plots displaying OR and 95% CI estimates for outcome in studies [28–30] evaluating anti-inflammatory therapies compared to placebo in patients who underwent surgical treatment due to cSDH. Squares represent the odds ratio; the bigger the square, the greater the weight given because of the narrower 95% CI. Diamond represents the odds ratio of the overall data.

Study or Subgroup	Experimental Events	Experimental Total	Control Events	Control Total	Weight	Odds Ratio M-H, Fixed, 95% CI
Jiang 2018	95	98	91	98	100.0%	2.44 [0.61, 9.71]
**Total (95% CI)**		**98**		**98**	**100.0%**	**2.44 [0.61, 9.71]**
Total events	95		91			

Heterogeneity: Not applicable
Test for overall effect: Z = 1.26 (P = 0.21)

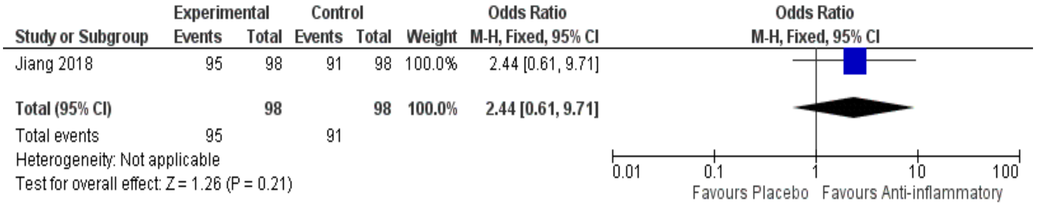

Supplementary Figure S3. Forest Plots displaying OR and 95% CI estimates for positive outcome in studies [31] evaluating anti-inflammatory therapies compared to placebo in patients with cSDH treated conservatively. Squares represent the odds ratio; the bigger the square, the greater the weight given because of the narrower 95% CI. Diamond represents the odds ratio of the overall data.

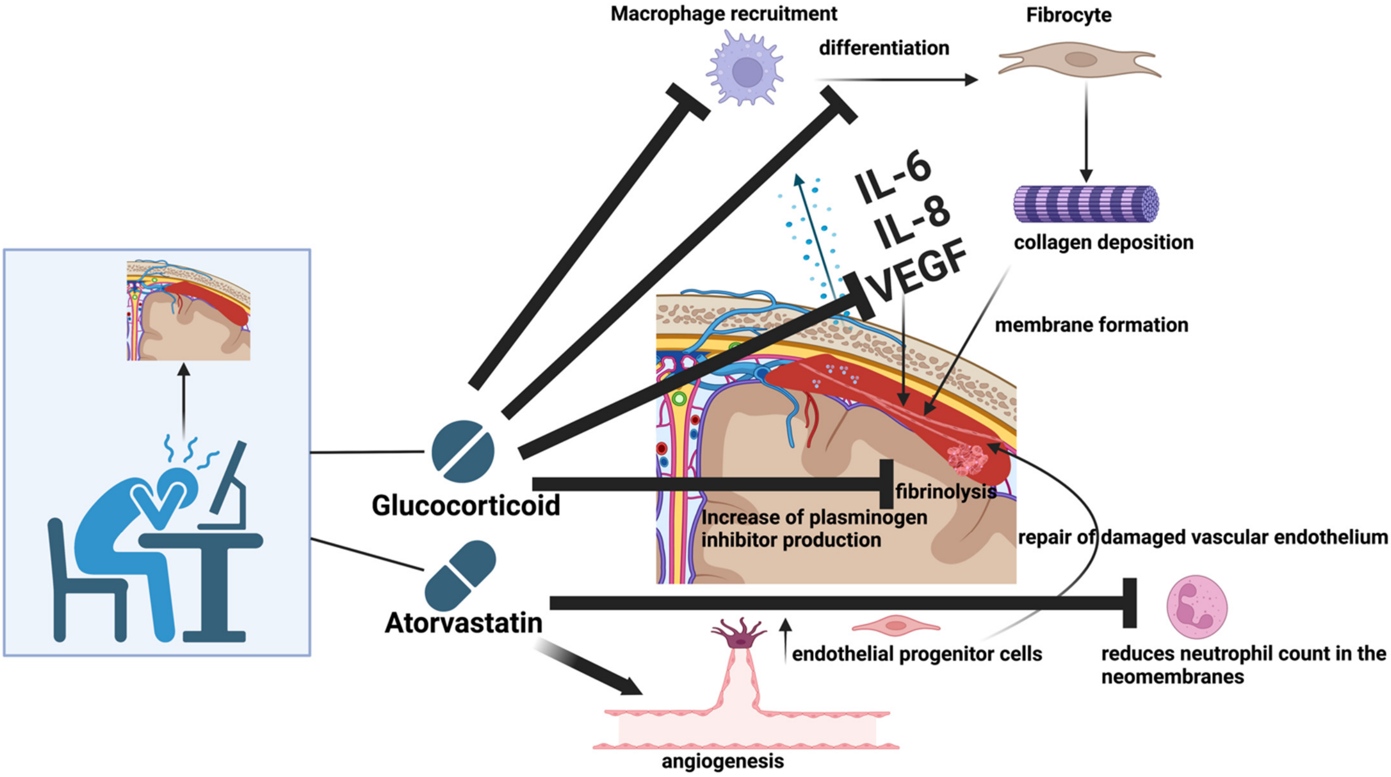

Supplementary Figure S4. Illustrative summary of potential anti-inflammatory functions of glucocorticoids or atorvastatin in cSDH. Glucocorticoids inhibit several inflammatory pathways such as the recruitment of inflammatory cells (e.g., macrophages), VEGF secretion, and macrophage differentiation. Atorvastatin stimulates the angiogenesis by increasing the level of endothelial progenitor cells in order to repair damaged vascular endothelium. Furthermore, atorvastatin acts anti-inflammatory by reducing the number of neutrophils in the neomembranes of cSDHs.
